# Reasons for poor blood pressure control in Eastern Sub-Saharan Africa: looking into 4P’s (primary care, professional, patient, and public health policy) for improving blood pressure control: a scoping review

**DOI:** 10.1186/s12872-021-01934-6

**Published:** 2021-03-04

**Authors:** Mende Mensa Sorato, Majid Davari, Abbas Kebriaeezadeh, Nizal Sarrafzadegan, Tamiru Shibru, Behzad Fatemi

**Affiliations:** 1 Department of Pharmacy, Arba Minch University College of Medicine and Health Sciences, P.O. Box 21, Arba Minch, Ethiopia; 2 Department of Pharmacoeconomics and Pharmaceutical Administration, Faculty of Pharmacy, Tehran University of Medical Sciences, Tehran, Iran; 3 Isfahan Cardiovascular Research Center, Cardiovascular Research Institute, Isfahan University of Medical Sciences, Isfahan, Iran; 4 College of Medicine and Health Sciences, Arba Minch University, Arba Minch, Ethiopia

**Keywords:** Reasons for poor hypertension control, Patient factors, Professional factors, Primary healthcare, Public health policy

## Abstract

**Aim:**

Hypertension control in Sub-Saharan Africa (SSA) is the worst (less than one out of ten) when compared to the rest of the world. Therefore, this scoping review was conducted to identify and describe the possible reasons for poor blood pressure (BP) control based on 4Ps’ (patient, professional, primary healthcare system, and public health policy) factors.

**Methods:**

PRISMA extension for scoping review protocol was used. We systematically searched articles written in the English language from January 2000 to May 2020 from the following databases: PubMed/Medline, Embase, Scopus, Web of Science, and Google scholar.

**Results:**

Sixty-eight articles were included in this scoping review. The mean prevalence of hypertension, BP control, and patient adherence to prescribed medicines were 20.95%, 11.5%, and 60%, respectively. Only Kenya, Malawi, and Zambia out of ten countries started annual screening of the high-risk population for hypertension. Reasons for nonadherence to prescribed medicines were lack of awareness, lack of access to medicines and health services, professional inertia to intensify drugs, lack of knowledge on evidence-based guidelines, insufficient government commitment, and specific health behaviors related laws. Lack of screening for high-risk patients, non-treatment adherence, weak political commitment, poverty, maternal and child malnutrition were reasons for the worst BP control.

**Conclusion:**

In conclusion, the rate of BP treatment, control, and medication adherence was low in Eastern SSA. Screening for high-risk populations was inadequate. Therefore, it is crucial to improve government commitment, patient awareness, and access to medicines, design country-specific annual screening programs, and empower clinicians to follow individualized treatment and conduct medication adherence research using more robust tools.

## Background

Hypertension is a major risk factor for cardiovascular diseases that significantly increases the risks of developing heart, brain, kidney, and other vascular diseases [1–3]. Globally less than 20% of people with hypertension have controlled their blood pressure [4]. Hypertension is responsible for at least 45% and 51% of deaths due to heart disease, and stroke, respectively [5]. The health system in SSA has the worst performance, with only 18–29.9% of participants received treatment, and 5–10.3% achieved control of their hypertension [6, 7]. In reality, it could be possible to achieve adequate blood pressure (BP) targets in about 70–80% of patients by improving adherence and/or intensifying drug therapy [8, 9].

Hypertension is the most extensively studied risk factors for cardiovascular disease [10–13]. However, its management and control are unsatisfactory. Studies indicated that the current prevalence is expected to double by 2030, unless health system, professionals, and SSA patients take steps to prevent and control hypertension. Additionally, moving from the current 7% BP control to WHO sustainable development goal target of 50% BP control and reducing NCDs' mortality by one-third in 2030 might be an ambition to the region [14–16].

Knowing the reasons for the worst blood pressure control in Eastern Sub-Sharan Africa will have several public health advantages. It will give direction for selecting and prioritizing interventions that can provide better patient outcomes for policymakers. For health care providers, it will provide information on the unmet needs of hypertension care. For patients, it will improve BP control and reduce hypertension associated morbidity and mortality. Finally, it will highlight areas that need further research to improve blood pressure control in sub-Saharan Africa for researchers. Several studies were conducted concerning hypertension care in SSA countries. Yet, none of them are comprehensive enough to provide a clear picture for policymakers, providers, primary healthcare systems, and patients concerning the reasons for the worst BP control in the region. Therefore, this scoping review was conducted based on PRISMA extension for scoping review protocol to identify and describe the possible reasons for poor blood pressure (BP) control based on 4Ps (patient, professional, primary healthcare system, and public health policy factors) among ten Eastern SSA countries [17, 18].

## Methods

### Data sources and search strategy

In eastern SSA, there is lack of comprehensive evidence on reasons of poor blood pressure control. Out of 20 Eastern SSA countries, we included ten countries in this review. Countries included were; Ethiopia, Kenya, Malawi, Mozambique, Rwanda, Seychelles, United Republic of Tanzania, Uganda, Zambia, and Zimbabwe [19, 20]. Countries are selected based on the following criteria: availability of recent national STEPS survey report, availability of primary health system capacity survey to provide care for patients with CVDs, availability of national chronic disease management strategies, accessibility of national health policy, and accessibility of health sector improvement strategies, directives, strategies, guidelines, and manuals.

### Search strategy

We searched articles written in the English language from January 2000 to May 2020 from the following databases: PubMed/Medline, Embase, Scopus, Web of Science, and Google Scholar with a systematic search query (available in Additional file 1). The following national documents are included in addition to systematically searched articles. These include the National health policy of selected countries, national chronic disease prevention, management and control strategies of selected countries, national quality improvement strategies, and national technology integration strategies.

### Study types

Systematic reviews, clinical trials, cohort studies, observational and cross-sectional studies related to uncontrolled blood pressure and associated factors.

### Inclusion and exclusion criteria

Systematic reviews, clinical trials, cohort studies, observational and cross-sectional studies related to uncontrolled blood pressure and associated (patient, professional, public policy, and political) factors among adults in Eastern Sub-Saharan Africa are included. Studies conducted before January 2000, short communications, and conference proceedings are excluded. Articles that are not related to uncontrolled blood pressure and associated (patient, professional, public policy and political) factors among adults in Eastern Sub-Saharan Africa are excluded.

### Study selection

**Fig. 1:**
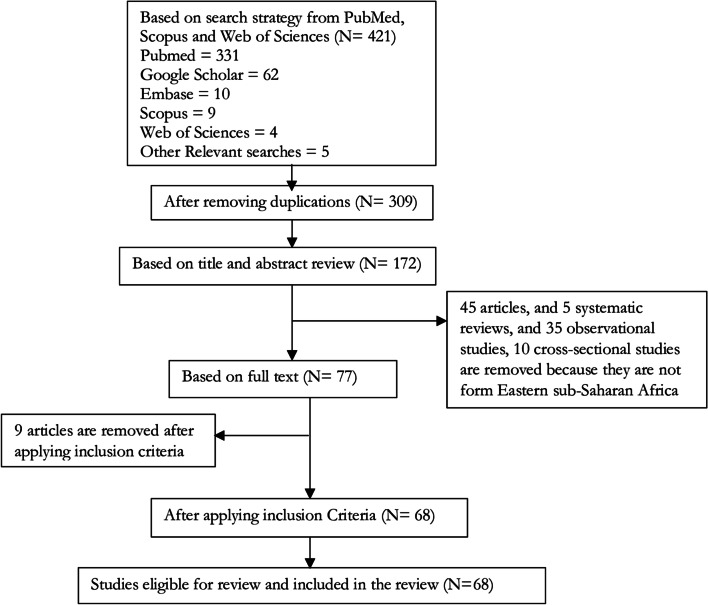
Flowchart representing the selection of sources of evidence and the number of articles excluded and eligible for review

From a total of 421 articles identified by the literature search, 172 potentially relevant articles were abstracted. After applying the inclusion–exclusion criteria listed above, 68 articles were found to be relevant (Fig. 1). Two investigators independently reviewed each study’s abstract against pre-specified inclusion and exclusion criteria. In case of disagreement on the article's quality, two authors discussed In front of the table in the third and fourth authors' presence.

### Data extraction

Two investigators abstracted population CVD risk factors, level of uncontrolled blood pressure, 4Ps (patient, professional, primary healthcare, and public policy related factors) associated with uncontrolled blood pressure data from all included studies. A second investigator checked these data for accuracy. Disagreements among us are managed through discussion in the presence of other authors.

### Data-items/variables

Prevalence of blood pressure control, patient-related, professional-related, primary healthcare system-related, and public health policy and politics, determinants of poor blood pressure control are used as data-items.

### Data synthesis and analysis

We qualitatively described and summarized the evidence on blood pressure control based on 4P’s (primary care, patient, professional, and public health policy-related factors). We also described the major primary healthcare challenges contributing to the region's worst BP control and strategies to address them.

## Results

### Hypertension care and risk factor control in Eastern Sub-Saharan Africa

Sixty-eight articles were included in this scoping review. The mean national prevalence of hypertension and BP control among adults in Eastern SSA was 20.95% and 11.55%. A majority of hypertensive were non-adherent to prescribed medications ranging from 25% Seychelles 94% Mozambique (Table 1) [21–39]. More than 80% of patients were taking < 5 servings of fruits and vegetables per day. The estimated prevalence of obesity ranges from 4 to 15%. The prevalence of obesity is relatively higher in Seychelles (15%) and Zimbabwe 12%. The lowest prevalence of obesity was recorded in Ethiopia and Uganda (i.e., 4% in adults ≥ 18 years) [21–39]. More than eight out of ten adults aged ≥ 18 years had at least one CVD risk factor [21–39].

**Table 1:** Prevalence of hypertension and related CVD risk factors in selected Eastern Sub-Saharan Countries

S.No	Country	Raised blood pressure, adults aged 18 + (%)	Proportion patients not taking antihypertensive drugs	Percentage of patients achieved BP control	Current tobacco smoking, adults aged 15 + (%)	Prevalence of obesity (BMI ≥ 30 kg/m^2^ in 18 + (%)	Physical inactivity, adults aged 18 + (%)	Harmful (heavy) alcohol consumption	Mean population salt intake, adults aged 20 + (g/day)	Parentage consuming fruits and vegetables < 5 servings/day
1	Ethiopia	16	71.6	< 30	4.0	4	14	12.4	6	97.6
2	Kenya	23.8	50.9		10	6	14	12.7	4.3	94.0
3	Malawi	22	94.9	1.5	12	5	14	7.7	4	97.5
4	Mozambique	23	50	Low	16	6	5	2	6	95.6
5	Rwanda	20	78	–	12.8	5	13.3	9	4	99.3
6	Seychelles	24	25	20.0	21	15	19	12	11	37.5%
7	Tanzania	25.9	77.1	3.1	14.1	7	7.5	9	7	97.2
8	Uganda	24.3	76.1	9.4	11	4	4.3	9	5	87.5
9	Zambia	20	80	6.7	15.8	7	20	5	6	90.4
10	Zimbabwe	20	–	–	11.8	12	25	5	8	–

S.No	Country	Availability essential NCD medicines	Raised blood glucose, adults aged 18 + (%)	Prevalence total cholesterol ≥ 5 mmol/L	Percentage with at least one risk factor	Proportion of 40–69 old with ≥ 30% CVD risk	Proportion of primary healthcare centres reported as offering CVD risk stratification	Having CVD guidelines that are utilized in at least 50% of health facilities	Availability essential technologies	References
1	Ethiopia	54.7	6	5.6	98.4	4.7	< 25%	0	50	[20–23]
2	Kenya	90	3.1	10.1	97	7.6	< 25%	0	66.7	[23, 24, 26–28]
3	Malawi	50	5.6	8.7	99	16.5	0	100	33.3	[23–25]
4	Mozambique	70	2.9	–	99.6	16.4	< 25%	0	66.7	[23, 24, 29]
5	Rwanda	100	3.1	2.6	99.6	16.35	0	100	83.3	[23, 24, 30]
6	Seychelles	100	10	36.7	–	–	< 25%	100	83.3	[23, 31]
7	Tanzania	60	9.1	4.4	82.6	16.6	< 25%	0	66.7	[23, 24, 32]
8	Uganda	50	3.3	6.7	–	–	< 25%	100	66.7	[23, 24, 33, 34]
9	Zambia	60	6.0	7.4	94.7	4.0	< 25%	100	66.7	[23, 24, 35, 36]
10	Zimbabwe	40	11.5	20.2	–	–	0	100	50	[23, 24, 37, 38]

Interventions to reduce body weight at the individual level often have little efficacy, except bariatric surgery. Therefore, weight control interventions should focus on the societal causes of the obesogenic environment and include interventions in all sectors (e.g. education, agriculture, finance, transports, nutrition, food industry, etc.) to enable people to choose healthier diets, including adequate food labeling, tax/subsidies on healthy/unhealthy foods; ban on advertising of unhealthy foods, healthy food in canteens in schools/workplaces. Multisectoral interventions should also address the structural environment to help people engage in more physical activity in their daily lives (e.g. bus/cycling lanes, safe sidewalks, promotion of public transports, disincentives to use private cars, etc.)

*CVD* cardiovascular disease, *NCD* non-communicable disease, *BP* blood pressure, *g/day* gram per day

The mean population salt consumption was 5.93 g/day, which is above the recommended daily allowance. It is also higher than WHO 2025 target (< 5 g/day) in most countries except Kenya 4.3 g/day, Malawi 4 g/day, and Rwanda 4 g/day [21–39]. The recommended dietary reference adequate intake (DRI/AI) level is 1500 mg of sodium for adults up to 50 years, 1300 mg for those 51 to 70 years, and 1200 mg for people with hypertension over 70 years [40]. Achieving a target of less than 5 g/day (≈ 2 g sodium) is expected to yield a 5.7% reduction of death from selected NCDs [41, 42].

Current tobacco consumption among adults + 15 years ranged from 4 to 21%. The highest smoking prevalence was recorded in Seychelles, 21%, followed by Mozambique, 16%, Zambia, 15.8%, and Tanzania, 14.1%. In contrast, the lowest prevalence of smoking was reported in Ethiopia, 4% [21–39]. The percentage of physical inactivity among adults + 18 years was highest in Zimbabwe 25%, Zambia 20%, and Seychelles 19%. The least physical inactivity was reported in Mozambique, 5% [21–39].

There is no CVD risk stratification at primary health centers in Malawi, Rwanda, and Zimbabwe. The cardiovascular disease risk stratification at the primary healthcare level was below 25% in other selected countries [21–39]. The availability of essential medicines for CVD care ranges from (40–100%). Availability of essential technologies for primary care suboptimal in most of the facilities. The availability of CVD guidelines at primary healthcare facilities was not ensured in four countries (Ethiopia, Kenya, Mozambique and Tanzania) [21–39].

The national NCD commitment and related progress report showed that most countries had the lowest progress, except Seychelles and Kenya. The major unaddressed areas were measures to reduce unhealthy diet followed by public awareness creation and annual screening for high-risk populations (Table 2) [43–53].

**Table 2:** Political commitment and national NCD related progress monitoring indicators and their status selected Eastern Sub-Saharan Countries

S. no.	Country	Ethiopia	Kenya	Malawi	Mozambique	Rwanda	Seychelles	Tanzania	Uganda	Zambia	Zimbabwe
1	National NCD targets	●	●	DK	●	○	●	●	○	○	○
2	Mortality data	○	○	○	○	○	●	○	○	○	○
3	Risk factor survey	◐	●	◐	◐	◐	◐	◐	●	◐	○
4	National NCD policy Strategy action plan	○	●	○	●	○	●	●	○	○	○
5	*Tobacco demand reduction measures*										
5.1	Increased excise taxes and prices	○	◐	NR	○	◐	◐	○	◐	◐	○
5.2	Smoke-free policies	◐	○	○	○	○	●	○	●	○	◐
5.3	large graphic health warnings/plain packaging	◐	◐	○	○	◐	●	○	◐	○	○
5.4	Bans on advertising, promotion and sponsorship	◐	●	○	◐	○	◐	◐	●	○	○
5.5	Mass media campaigns	◐	●	NR	○	NR	●	NR	○	○	○
5.6	Tobacco control (MPOWER score)	18	23	12	18	10	–	16	19	15	14
6	Harmful use of alcohol reduction measures:										
6.1	restrictions on physical availability	◐	◐	●	◐	◐	◐	◐	◐	◐	◐
6.2	advertising bans or comprehensive restrictions	◐	◐	○	○	○	◐	○	○	○	○
6.3	increased excise taxes	◐	◐	○	○	◐	◐	◐	◐	◐	◐
6.4	SAFER strategy implementation	◐	◐	◐	◐	◐	◐	◐	◐	◐	◐
7	*Unhealthy diet reduction measures*										
7.1	Salt/sodium policies	○	○	○	○	○	○	○	○	○	NR
7.2	Saturated fatty acids and trans-fats policies	○	○	○	○	○	○	○	○	○	○
7.3	Marketing to children restrictions	○	○	○	●	○	○	○	○	○	DK
7.4	Marketing of breast-milk substitutes restrictions	○	●	◐	●	◐	◐	●	●	◐	●
7.5	RPLACE strategy implementation	○	○	○	○	○	○	○	○	○	○
8	Public education and awareness campaign on physical activity	○	○	○	○	○	●	○	○	●	DK
9	Guidelines for management of cancer, CVD, diabetes and CRD	●	◐	●	○	●	◐	○	●	●	●
10	Drug therapy/counselling to prevent heart attacks and strokes (%)	11.5	6	12	○	○	○	38	14	13	○
11	Annual screening campaign for CVD risk factors	○	○	○	○	◐	○	○	○	◐	○
	References	[41–51]									

◐, partially achieved; ●, fully achieved; ○, not achieved; DK, don’t know; NR, No Response

MPOWER: Monitoring tobacco use and prevention policies, Protecting people from tobacco smoke, Offering help to quit tobacco use, Warning people about the dangers of tobacco, Enforcing bans on tobacco advertising, promotion, and sponsorship, and Raising taxes on tobacco

SAFER: Strengthen restrictions on alcohol availability, Advance and enforce drink driving counter measures, Facilitate access to screening, brief interventions and treatment, Enforce bans or comprehensive restrictions on alcohol advertising, sponsorship and promotion, and Raise prices on alcohol through excise taxes, and pricing policies

REPLACE: Review dietary sources of industrially-produced trans-fat and the landscape for required policy change. Promote the replacement of industrially-produced trans-fat with healthier fats and oils. Legislate or enact regulatory actions to eliminate industrially-produced trans-fat. Assess and monitor trans-fat content in the food supply and changes in trans-fat consumption in the population. Create awareness of the negative health impact of trans-fat among policy-makers, producers, suppliers, and the public. Enforce compliance with policies and regulations

### Primary healthcare capacity in Eastern Sub-Saharan Africa

**Fig. 2:**
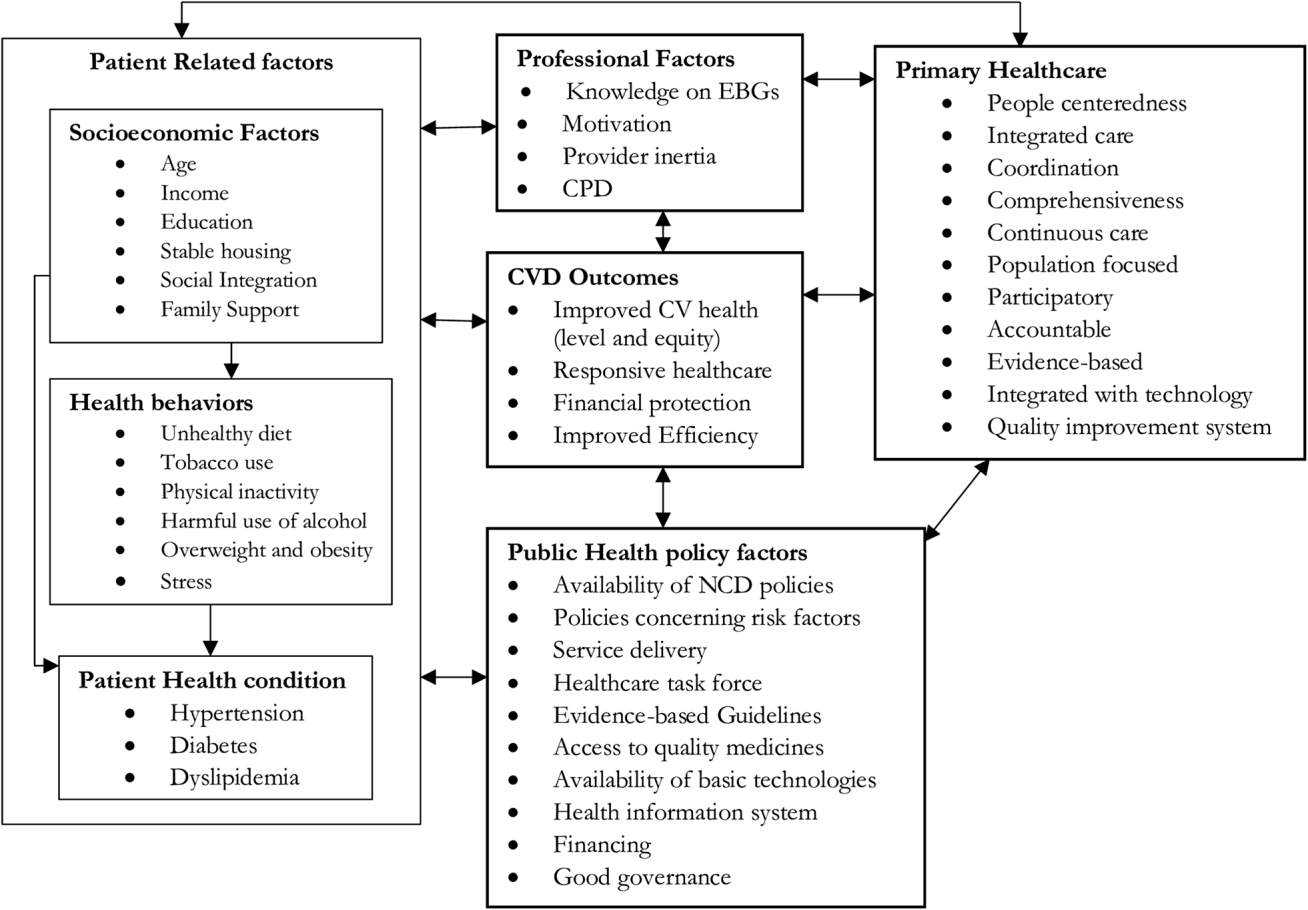
Understanding 4Ps and cardiovascular disease outcome. Adapted from different literatures

Before the development of fatal and debilitating complications of CVD, there is a long latency period. This is because atherosclerosis begins early in life and progresses gradually through adolescence and early adulthood with no symptoms. This long latency period can be described by the complex set interactions involving 4Ps (patients, professionals, primary healthcare, and public health policy) in the CVD care continuum (Fig. 2) [40, 54–60].

A Comprehensive approach for hypertension targeting total risk approach is vital for reducing CVD related morbidity and mortality [16]. Planning for providing comprehensive healthcare to society should move beyond the clinical care arena. As evidenced by consideration of the 11 other sustainable development goals by WHO member states [61].

Primary healthcare (PHC) is a basic pillar for management and control of hypertension. Service availability and readiness (SAR) for major NCDs at health facilities in Ethiopia showed that facility readiness for management of diabetes and CVD were 22% and 41% respectively [62, 63]. A SAR survey in Kenya showed that only 34% of primary healthcare facilities are ready to provide NCD services [64]. In Tanzania, SAR survey showed that only 28% of the assessed facilities were prepared for the outpatient primary care of hypertension. About 9% and 42% of the assessed facilities reported having at least one trained staff and guidelines for hypertension [65, 66]. A similar study from Zambia showed that only 13.1% of facilities were ready to manage NCDs [67].

Assessment of care provision for hypertension at the emergency department of an urban hospital in Mozambique showed critical gaps in health facility readiness to address hypertension. The care pathway is not simplified, and there was no hypertension risk stratification algorithm. The average availability of medicines was 28% [68]. A service readiness of health facilities in Kenya, Malawi, Rwanda, Uganda, and Tanzania showed that the mean values for the service readiness index were 77% for hospitals and 52% for health centers [69].

The capacity survey in Uganda showed that 92.9% of facilities reported managing patients with hypertension. Forty-six percent of the facilities had guidelines for managing hypertension, and 28% did not have stethoscopes. About half of the facilities had antihypertensive medicines in stock [70, 71]. A similar survey from Zimbabwe showed that about 68% and 61% of facilities managed CVD and diabetes, respectively [72]. A study conducted in Malawi showed that 20% of hospitals and 72% of health centers had copies of NCDs' clinical guidelines for NCDs. Overall less than 7.5% of drugs were available at all sites [73].

A service readiness and availability survey conducted in Seychelles showed that the mean score of basic amenities, essential equipment, and infection prevention was 88% and above, whereas the scores for diagnostic capacity and essential medicines were 41% and 61%, respectively. Access to services is high in Seychelles: travel time to a health facility is less than 30 min for more than 75% of patients, and waiting time is less than 30 min for 70% [74].

Only three out of ten included countries, Kenya [75], Malawi [76], and Zambia [77] have an annual screening program for high-risk patients. The introduction of country-specific annual screening systems is critical for early detection and management of hypertension. These annual screening sites can be managed by available health resources. For example, 'May is a Measuring Month' experience from Malawi [76] and Zambia [77], and unused health taskforce through efficient management.

### Patient-related factors for poor BP control

About 80% of global CVD related deaths were occurring in LMICs. The poor have the worst outcomes from CVD, largely due to a lack of access to preventive services and ongoing treatments [78, 79]. According to WHO, the African region has the highest prevalence of hypertension, and the majority of patients are unaware of their hypertensive status [80, 81]. A systematic review of the burden of hypertension in SSA showed that only 27% of the hypertension patients were aware of their hypertensive status [15]. A study conducted in Ethiopia showed that lack of awareness of hypertension and related complications, nonadherence to a healthy life style, middle age, and old age were significant predictors of uncontrolled hypertension [82].

A study conducted in northern Tanzania showed that 48.3% of the participants were aware of their disease. Still, almost all (95.3%) had uncontrolled hypertension [83]. Good knowledge, attitude, and practices concerning hypertension were independently associated with increased BP control, even after adjusting for mediation through adherence [84].

A study from Uganda showed that awareness about hypertension was 28.2%, and only 9.4% of participants had controlled their BP [35]. A study conducted in Kenya showed that about 53.6% of patients believed they should stop taking antihypertensive medication once hypertension is controlled [85]. A study from Malawi showed that 62% of the hypertensive patients were aware of their blood pressure [86]. A study from Tanzania showed that awareness about hypertension was below 10% [84, 87].

Low adherence to treatment is the main reason for poor BP control [88]. According to facility-based studies in Ethiopia, medication adherence was 61.8–75.1% [89, 90]. Similar research conducted in Kenya showed that 64% of patients had missed medication. Age, religious beliefs, and cost of medication were independent predictors of nonadherence [91]. Zimbabwe's study showed that good adherence and having received health education on hypertension were protective against uncontrolled hypertension [92].

A cross-sectional study conducted in Tanzania showed that the proportion of participants with treatment compliance was 56%. Age ≥ 64 years, being female, being married, perceived susceptibility, and the perceived benefit was statistically associated with treatment adherence [93]. A study assessed factors influencing treatment adherence in Malawi showed that 66.7% of the patients using the clinic’s services did not adhere to their treatment. Predictors for nonadherence were long waiting times at the clinic and the interrupted supply of medicines [94].

A systematic review in LMICs showed patient socioeconomic factors as the major reason for nonadherence [95]. Another study from twelve SSA countries showed that using traditional medicine and individual wealth index as an independent predictor of poor adherence to medication [96]. A cross-sectional study conducted in Uganda showed that only 17% were adherent to antihypertensive medications. The main causes of nonadherence were lack of knowledge and lack of prescribed drugs [97]. A similar study conducted in Zimbabwe showed that self-reported drug adherence was 40.2%. Participants with normal BP measurements were more than three times as likely to report maximal adherence to prescribed drug schedules [98].

A study conducted in Zimbabwe showed that the treatment default rate was 30.9%, and 25% of respondents on medication did not know their blood pressure control status. Knowledge related to hypertension was poor. The educational status of respondents was an independent predictor of knowledge about hypertension [99].

A facility-based cross-sectional study conducted in Addis Ababa showed that 59.9% of the patients have uncontrolled blood pressure. Nonadherence, obesity, family history, excessive salt consumption, and presence of comorbidity were associated with uncontrolled blood pressure [100]. The affordability of drugs has sometimes been implicated in poor treatment adherence. Higher co-payment, medication side effects, and poor patient-provider relationship were associated with poor adherence [9, 101]. Adherence to a healthy lifestyle is equally or probably more important for BP control [102]. However, more than 80% of adults in eastern SSA were taking < 5 servings of fruits and vegetables per day [21–39].

### Provider related factors affecting BP control

Hypertension treatment guidelines state explicitly that most hypertensive patients will require two or more drugs to achieve BP control [103]. Health care providers’ practice habits, particularly the reluctance to intensify treatment and therapeutic inertia, have been implicated in the failure to meet BP goals [9]. Physician uncertainty over the patient’s usual BP, adherence, or value of continuing efforts to control BP through lifestyle changes is the main contributor to clinical inertia [104].

Another important provider related barrier to BP control was knowledge of evidence-based guidelines. Health facility capacity to manage hypertension in the Uganda survey showed the need of additional training on hypertension management by all health workers [70]. A similar study from Zimbabwe showed that professional knowledge was poor among 47.7% of health workers [105, 106]. Another study from Rwanda showed that 43% of clinicians had poor knowledge [107]. Similar study from northern Tanzania showed that poor point-of-care communication, poor understanding of hypertension and structural barriers such as long wait times and undertrained providers were barriers to optimal care [83].

### Public health policy and political factors

The availability and affordability of essential medicines and diagnostic technologies for CVD in Eastern SSA were barriers to BP control. This could be due to lack of political will, insufficiency of human resources or funding, conflict of interests, and weak social insurance system [108, 109]. A recent study showed that combination (aspirin, β-blocker, ACE inhibitor, and statin) for the secondary prevention of CVD was not affordable for 60% of households [110]. According to Ethiopia's third national pharmaceutical sector survey, none of the essential medicines for NCDs were affordable [63].

Poor accessibility of health services is also contributing to poor BP control. In Ethiopia, living within 30 min distances of a public-sector hospital was associated with improved adherence to therapy [111]. A recent household study showed that in Uganda, 35% of households had to travel > 15 min to reach a health facility and only 16% of Ugandan households have access to medicines for NCDs [112].

In many LMICs, there is a wide gap between evidence-based recommendations and current practice. Standard treatment protocols are important to improve quality, reduce variability, and simplify the treatment options. According to the WHO country capacity survey of 2015, 67% of countries reported having evidence-based national guidelines for CVD management [113]. A retrospective cohort study conducted in Ethiopia showed that treatment was intensified for only 23% of patients with uncontrolled BP [114]. A similar study from Kenya showed that treatment guideline adherence for stage two hypertension was 75% [115].

The study evaluated the impact of poverty on hypertension and CVD in SSA showed that about 24% of the world's disease burden is in Africa, but only 3% of the world’s healthcare workers and just 1% of the global financial resources to manage this burden. Thirty-six out of 57 countries that can’t meet an accepted basic healthcare standard are in SSA [116].

The study evaluated the impact of urbanization and international trade and investment policies as determinants of NCDs in SSA showed that urbanization carries potential health benefits due to improved access to foods. However, this has caused an increased reliance on cheap, highly processed food commodities. Lucrative business advantage from such foods has promoted the creation of ‘obesogenic’ environments, which, through progressively integrated global food systems, have been increasingly ‘exported’ to developing nations [117].

Maternal and child malnutrition in low and middle-income countries encompasses undernutrition and a growing problem with overweight and obesity [118]. The continued undernourishment prevalence rate of 20–30% in SSA, alongside an increasing obesity epidemic, is alarming. This is because entering into a rapid nutrition transition, wherein fetal adaptations to an early nutrient-limited environment increase NCDs' later life susceptibility [119–121].

Chronic nutritional deficit during prenatal and continuing in postnatal life leads to energy conservation. This aim of energy conservation increases the risk of obesity and hypertension later in life. The prevalence of hypertension among stunted adolescents was higher than non‐stunted 21% versus less than 10%. The prevalence of hypertension in undernourished preschool children, or those who recovered from undernutrition, was higher than that in controls 29%, 20%, and 2%, respectively [122].

Race and ethnicity are becoming essential concerns for hypertension treatment and control. Compared with white patients, hypertension in black patients tends to be more common, starting early, more severe, and rapidly progressive [123]. Black patients are at significantly greater risk for stroke than white patients, especially at younger ages. For example, the adjusted relative risk of stroke is more than two times higher in hypertensive black patients aged 45–64 years [124]. The Important risk factors for hypertension among black patients include lower socioeconomic status, high-salt intake, and poor maternal nutrition [125, 126].

## Discussion

In this scoping review, we addressed hypertension care and reasons for the worst BP control among 10 Eastern SSA courtiers from a 4P (patient, provider, primary healthcare, and public health policy and politics) perspective [21–39]. The mean national prevalence of hypertension and the level of BP control among adults + 18 years in 10 Eastern SSA countries was 20.95% and 11.55%, respectively [21–39]. In reality, it is possible to achieve effective BP targets in about 70–80% of patients by improving adherence and/or intensifying drug therapy [8, 9]. Lack of early detection, inadequate treatment, and poor treatment adherence were the main contributors to poor BP control. These main contributors can be described from 4Ps (patients, professionals, Primary healthcare system, and Public health policy) perspective [14, 15, 123, 124].

Only three countries (Kenya, Malawi, and Zambia) started annual screening in 2017 [75–77]. The recent systematic review conducted in SSA showed that only a small proportion of the people with hypertension were aware of their hypertensive status [15]. Therefore, ‘a May is Measurement Month’ experience of Kenya, Malawi, and Zambia can be expanded to other countries like Ethiopia. Looking for new healthcare task force opportunities will have paramount importance to minimize the possible costs and enhance feasibility. For example, in Ethiopia, graduating class health science students have an annual community deployment program before graduation. The integration of yearly screening of high-risk patients for hypertension can be done with a slight orientation on patients’ comprehensive screening for cardiovascular disease based on a total risk approach.

High political commitment and downward accountability culture from these countries’ governments are needed to improve access to necessary technologies and essential drugs for managing hypertension to cope with associated demand increase due to the annual screening program [61, 74]. In addition to this, the capacitation of primary healthcare workers in the front line for hypertension care through training or continuous professional development is important to improve hypertension management and BP control. In this regard, a practical approach to care kit (PACK) clinical decision support tool designed to simplify, standardize, and strengthen primary healthcare delivery in LMICs can be helpful [127].

The great majority of hypertensive patients in Eastern SSA were non-adherent to medications prescribed by healthcare professionals. For example, 94.9%, 78%, and 82.0% were not taking anti-hypertensive medications in Malawi, Rwanda, and Zambia [21–39]. Determinants of nonadherence include patient-related factors (e.g., low health literacy and weak involvement in treatment decision-making), physician-related factors (e.g., polypharmacy complex regimens, ineffective communication, and uncoordinated care by multiple physicians), and healthcare systems related (e.g., short consultation time, limited access to care and lack of health information technology) [128–131].

Treatment adherence is not directly translated to BP control in most countries included in this review [9, 132]. The revealed inconsistency between adherence and BP control suggests three things. The first is how treatment adherence is measured. This is because treatment adherence should be evaluated comprehensively (i.e., medication and healthy lifestyles) [40]. The second is what methods are used to measure adherence. Most of the adherence studies stated self-reported adherence. It is important to consider more robust adherence measurement tools, like therapeutic drug monitoring for uncontrolled BP patients taking an intensified dose of three drugs [132]. The third point is the quality of medicines being used for the management of hypertension. For example, in Rwanda, focusing on cardiovascular medicines showed two of 10 products purchased from private outlets were substandard [133]. Similarly, counterfeit and sub-standard drugs were reported in the Ethiopian pharmaceutical supply chain [134]. Poor access to medicines, weak technical capacity, poor pharmaceutical sector governance, and low community awareness are backbones for drug trafficking [135].

Hypertension related awareness and knowledge is low in Eastern SSA. Enhancing individuals' and communities' health literacy is an important measure to improve the acceptability of NCD interventions and BP control [16, 40, 136–138]. Patients who have better knowledge about the disease and its treatment outcomes were more likely to adhere to treatments prescribed by professionals [15, 35, 82–87]. The medication adherence club experience form Kenya can be contextualized and applied in other countries. The approach has reduced loss to follow-up and supported burden reduction and flexibility of regular clinical review for patients [139].

Lack of access to medicines for hypertension is one of the major barriers to hypertension care in Eastern SSA. It can be addressed by improving medicine financing, selection process, manipulating the procurement process for efficiency [133, 140], removing taxes and duties on essential medicines and control markups, improving the health insurance system, and providing incentives for pharmaceutical manufacturers to invest in quality medicine production. Accessibility of health services can be addressed by increasing operational hours of clinics providing free or subsidized care, decreasing waiting times by streamlining organizational processes and changes in regulations, and increasing the perceived quality of care [133, 140].

Quality of medicines can be ensured by strengthening the capacity of the National medicines regulatory authorities, creating a business environment that is favorable for the private sector to invest in secure supply chains, regular quality testing at procurement and sales sites, and consumer short message service (SMS) and mobile application verification of product authenticity [133, 140]. The availability of evidence-based guidelines is essential for care standardization and improving patient outcomes. Therefore, it is essential to ensure their availability in both public and private health facilities. However, in Eastern SSA, guideline availability is only concerned in public health facilities. Patients may use private facilities for their NCD management or medication refill due to accessibility, affordability, or medicines availability in private facilities.

Adherence to lifestyle factors was poor in Eastern SSA. Adherence to health behaviors is highly dependent on public health policy related variables [141]. Therefore, it is critical to enact strong specific laws and follow the implementation of the laws to reduce cardiovascular disease-related complications. All countries included in this review have no specific laws against unhealthy diet consumption. Almost all have partially implemented laws against tobacco smoking, physical activity, and harmful alcohol [21–39].

Poor diet killed more people globally than tobacco and high blood pressure [102, 142]. The majority of peoples in eastern SSA were farmers. However, more than 80% of patients took < 5 servings fruits and vegetables per day [21–39]. Therefore, the modernization of farming, empowering farmers to produce more fruits and vegetables, and increasing awareness about a healthy diet for the general public will have a paramount contribution to change the very low consumption of fruits and vegetables in the region.

The globalization of unhealthy lifestyles (smoking, high-fat diets, salt consumption, and alcohol use) is a political and trade issue. Therefore, enacting strong policies with priority to citizens' health, re-evaluating trade policies, and agreements and collaborating and controlling industries working in this area is critical. It is also equally important to ensure transparency of strategies and actions to control business owners' big hands in the area, as it is a source of billion dollars investment [117].

## Conclusion

The rate of BP treatment and control and medication adherence was very low in Eastern SSA. The low treatment rate is mainly due to under-diagnosis and lack of screening service for a high-risk population. Poor adherence to prescribed medicines was related to patients, professionals, the primary healthcare system, and public health policy. In addition to this, medication adherence is not directly translated to BP control. Adherence to healthy lifestyles was also extremely low. Eight out of 10 adults aged ≥ 18 years above had at least one cardiovascular disease risk factor, indicating awaiting future burden of NCDs in the region. The worst BP control reasons were poor treatment adherence, lack of early screening, weak political commitment, and socioeconomic factors, including maternal and child malnutrition.

Based on this scoping review findings, we provide the following recommendations for Eastern SSA countries to improve BP control. (1) Improving government commitment to achieving sustainable development goal 3.4, as evidenced by weak political commitment. (2) Improving patient awareness through facility-based health education or mass media. (3) Improving access to essential medicines and necessary CVD care technologies. (4) Ensuring availability of evidence-based guidelines in both public and private health facilities. (5) Improving fruit and vegetable consumption through improving awareness about nutrition for heart health. (6) Designing country-specific annual screening systems for high-risk patients. (7) Evaluating trade policies in light of citizens' health is a priority to reduce the import of obesogenic foods. (8) Conducting medication adherence research by using more strong tools for patients with uncontrolled BP after appropriate dose intensification.

## Supplementary Information

**Additional file 1.** Search strategy.

## Data Availability

This is a scoping review, and we have used only published articles. The search strategy is provided in the supplementary file.
