# Supplementary material for: Reasons for poor blood pressure control in Eastern Sub-Saharan Africa: looking into 4P’s (primary care, professional, patient, and public health policy) for improving blood pressure control: a scoping review
Source: BMC Cardiovasc Disord. 2021 Mar 4;21:123. doi: 10.1186/s12872-021-01934-6 (PMC7971125; doi:10.1186/s12872-021-01934-6)
Supplement: Supplementary file 1 — Additional file 1. Search strategy. [file 12872_2021_1934_MOESM1_ESM.docx]

**Literature Search Strategy**

**Pubmed/Medline:** ((((((("health"[MeSH Terms] OR "health"[All Fields]) AND ("drug delivery systems"[MeSH Terms] OR ("drug"[All Fields] AND "delivery"[All Fields] AND "systems"[All Fields]) OR "drug delivery systems"[All Fields] OR "system"[All Fields]) AND factors[All Fields]) OR (("patients"[MeSH Terms] OR "patients"[All Fields] OR "patient"[All Fields]) AND factors[All Fields])) OR (professional[All Fields] AND factors[All Fields])) OR (("policy"[MeSH Terms] OR "policy"[All Fields]) AND factors[All Fields])) AND associated[All Fields]) AND (Uncontrolled[All Fields] AND ("blood pressure"[MeSH Terms] OR ("blood"[All Fields] AND "pressure"[All Fields]) OR "blood pressure"[All Fields] OR "blood pressure determination"[MeSH Terms] OR ("blood"[All Fields] AND "pressure"[All Fields] AND "determination"[All Fields]) OR "blood pressure determination"[All Fields] OR ("blood"[All Fields] AND "pressure"[All Fields]) OR "blood pressure"[All Fields] OR "arterial pressure"[MeSH Terms] OR ("arterial"[All Fields] AND "pressure"[All Fields]) OR "arterial pressure"[All Fields] OR ("blood"[All Fields] AND "pressure"[All Fields])))) AND ("africa south of the sahara"[MeSH Terms] OR ("africa"[All Fields] AND "south"[All Fields] AND "sahara"[All Fields]) OR "africa south of the sahara"[All Fields] OR ("sub"[All Fields] AND "saharan"[All Fields] AND "africa"[All Fields]) OR "sub saharan africa"[All Fields]) AND ("has associated data"[Filter] AND ("2000/01/01"[PubDate] : "2020/12/31"[PubDate]))

**Web of sciences**: (((((((health system factors) OR patient factors) OR professional factors) OR policy factors) AND associated with) AND Uncontrolled blood pressure) AND Sub-Saharan Africa)

**Scopus**: health AND system AND factors OR patient AND factors OR professional AND factors OR policy AND factors AND associated AND with AND uncontrolled AND blood AND pressure AND sub-Saharan AND africa

**Embase**: (((('health'/exp OR health) AND system AND factors OR 'patient'/exp OR patient) AND factors OR professional) AND factors OR 'policy'/exp OR policy) AND factors AND associated AND with AND uncontrolled AND ('blood'/exp OR blood) AND ('pressure'/exp OR pressure) AND 'subsaharan' AND ('africa'/exp OR africa)

**Google scholar:** health AND system AND factors OR patient AND factors OR professional AND factors OR policy AND factors AND associated AND with AND uncontrolled AND blood AND pressure AND Sub-Saharan AND Africa
